# Introducing a novel prediction index for treatment quality of gamma knife and stereotactic‐based radiotherapy: Nabaa Efficiency Index ($\eta^{\mathrm{Nabaa}}$)

**DOI:** 10.1002/acm2.70805

**Published:** 2026-10-06

**Authors:** Nabaa Mohammed Alsiraj, Haydar Hamza Alabedi, Mustafa Salih Almusawi, Moneer K. Faraj, Rozilawati Ahmad, Sura Abdulkareem Madlool Alzuabidi

**Affiliations:** ^1^ Programme of Diagnostic Imaging and Radiotherapy Faculty of Health Sciences Universiti Kebangsaan Malaysia Kuala Lumpur Malaysia; ^2^ Gamma Knife Center Ghazi Alhariri Hospital for Specialist Neurosurgeries, Ministry of Health Baghdad Iraq; ^3^ Surgery Department College of Medicine, Baghdad University Baghdad Iraq; ^4^ Surgery Department College of Medicine University of Warith Al‐Anbiyaa Karbala Iraq; ^5^ Warith International Cancer Institute Karbala Iraq; ^6^ Medical Physics Department Faculty of Medical Sciences Jabir Ibn Hayyan Medical University Al‐Kufa Iraq; ^7^ Al Amal National Hospital for Oncology, Medical Complex Baghdad Iraq

**Keywords:** efficiency index, Nabaa Efficiency Index, Paddick conformity index (PCI), RTOG

## Abstract

**Background:**

An accurate assessment of the radiotherapy plans requires an accurate dose to the target and efficient confinement of the doses. However, present indices do not completely cover these aspects in a single framework.

**Purpose:**

To develop a unified, dimensionless metric that integrates geometric conformity and dosimetric efficiency for evaluating stereotactic and fractionated radiotherapy plans aims to reduce QA workload by providing a rapid, quantitative estimate of plan acceptability, allowing early prediction of treatment plan performance.

**Methods:**

In this study, the Nabaa Efficiency Index $\eta^{Nabaa}$ is introduced and defined as the product of the Conformity Index (CI) and a Dose‐Efficiency Ratio (DER), evaluated at 50% and 90% isodose levels ($\eta_{50\%}^{Nabaa}$, $\eta_{90\%}^{Nabaa}$). We analyzed theoretical behavior against normalized dose–volume ratios and compared $\eta^{Nabaa}$ with established indices of conformity, homogeneity, and the Paddick efficiency index $\left(\eta_{50\%}^{Paddick}\right)$. Clinical validation used 100 plans (50 Gamma Knife, 50 VMAT‐SRS). Statistics included ANOVA, correlations, ROC/AUC, and decision‐curve analysis.

**Results:**

$\eta_{50\%}^{Nabaa}$ and $\eta_{90\%}^{Nabaa}$ were monotonic, bounded, and dimensionless. $\eta_{50\%}^{Nabaa}$ distinguished gradient/halo efficiency between stereotactic and linac plans (*p* < 0.001). $\eta_{90\%}^{Nabaa}$ most strongly reflected prescription‐dose conformity (pooled AUC = 0.94) and outperformed the Paddick Efficiency Index $\left(\eta_{50\%}^{Paddick}\right)$ in discrimination and interpretability.

**Conclusion:**

$\eta^{Nabaa}$ unifies geometric and dosimetric assessment across modalities. Its simplicity, physical interpretability, and software‐ready formulation support integration into automated QA and treatment‐planning pipelines.

## 1 INTRODUCTION

Radiotherapy plays a pivotal role in the treatment of cancerous (malignant) and benign tumors. Since the introduction of Gamma Knife radiosurgery by Lars Leksell in the late 1960s, radiotherapy has undergone continuous technological advancement. These developments have led to highly conformal treatment techniques capable of delivering ablative doses with increasing geometric precision and improved normal tissue sparing. These devices utilized radiative sources like cobalt‐60 to treat tumors and functional disorders, primarily trigeminal neuralgia, thereby ushering in a new era in radiotherapy techniques. Contemporary linac‐based modalities, such as those using Volumetric Modulated Arc Therapy (VMAT) and Intensity‐Modulated Radiotherapy (IMRT), have brought radiotherapy to the level of personalized and highly conformal discipline. Nevertheless, despite these advances, there remains an age‐old problem in the field of clinical medical physics,^1^, ^2^, ^3^ i.e., the development of standardized, quantitative, and universal methodologies to assess plan quality.

Geometric conformity and dosimetric efficiency are the two most important elements used to traditionally define the plan quality in radiotherapy. Geometric conformity is used to measure the extent to which the isodose surface that has been given matches the target volume and, as a result, provides spatial accuracy in dose delivery. Dosimetric efficiency, on the other hand, is an indicator of how efficiently the energy delivered is used in the target, sparing proximate normal tissues. The balance between the two parameters determines the therapeutic ratio and hence the probability of tumor control (TCP) and complication in normal tissues (NTCP). The Radiation Therapy Oncology Group (RTOG) Conformity Index and the Paddick Conformity Index (PCI) are the most used metrics for assessing geometric conformity. The RTOG index is calculated as the simple ratio of the prescription volume and the target volume; it, however, ignores the internal dose heterogeneity and the dose spill. Paddick (2000) ^2^ has addressed these shortcomings with a geometric overlap formulation that provides better spatial accuracy by accounting for the variations in dose distribution within the target volume and minimizing the impact of dose spill on surrounding healthy tissues.

The Gradient Index (GI), Homogeneity Index (HI), and other parameters based on dose volume histograms (DVH) give a picture that is useful but not complete.The GI describes the steepness of dose fall‐off, while the HI measures the uniformity of dose in the target; however, neither measure assesses energy usage nor allows for a direct comparison between stereotactic and fractionated plans. The lack of a single, dimensionless index that characterizes both the geometric accuracy and dose efficiency draws attention to the need for a more complex analytical system.

Existing plan quality indices may be broadly divided into geometric indices (e.g., RTOG CI and Paddick Conformity Index (PCI)), gradient‐based indices (e.g., GI), and DVH‐derived parameters. While these metrics have given some useful, but isolated, information about the quality of plans, none of them simultaneously includes both the spatial conformity and the dose‐energy distribution within a single dimensionless framework. Previous attempts, such as the Paddick efficiency index, are geometry‐driven, focusing primarily on the geometric aspects of treatment plans without considering the dosimetric factors that are crucial for comprehensive evaluation. Therefore, a unified metric incorporating both geometric and dosimetric components remains lacking.

Modern radiotherapy encompasses a wide spectrum of treatment approaches, ranging from stereotactic radiosurgery (SRS) and stereotactic body radiotherapy (SBRT) to conventionally fractionated IMRT and VMAT. Although these techniques differ in prescription strategy, dose gradient, and treatment delivery, they all require reliable metrics for objective assessment of plan quality.^1^, ^3^

We propose a generalized Nabaa Efficiency Index $\eta^{Nabaa}$ framework that extends existing efficiency‐based plan evaluation by incorporating multiple prescription‐isodose levels within a unified mathematical formulation.

There have been two formulations that are defined to suit different treatment paradigms:

- **η_50_^Nabasa^**, emphasizing spatial confinement and gradient sensitivity using the 50% prescription isodose volume (PIV_50_), suitable for SRS and SBRT, and
- **η_90_^Nabaa^**, emphasizes prescriptive conformity using the 90% prescription isodose volume (PIV_90_), which is relevant for conventional fractionated or hypofractionated plans.

The dual‐index approach enables the use of one mathematical model to evaluate the efficiency of high‐dose ablative treatments and fractionated modalities, including VMAT and IMRT. Consequently, $\eta^{Nabaa}$ frameworks address the gap in understanding the two plan evaluation systems—stereotactic and conventional—and offer a dimensionless, cross‐cutting measure derived solely from analytical principles.

To address the limitations of existing indices, the following section details the mathematical derivation and validation of the proposed Nabaa Efficiency Index ($\eta^{Nabaa}$).

Subsequently, the primary objective of this paper is to develop a new index formula that is more generalized and accurate, encompassing the evaluation of conventional planning techniques such as IMRT and VMAT, as well as stereotactic radiotherapy, whether it is single‐fraction or fractionated.

## 2 METHODOLOGY

### 2.1 Mathematical formulation of Nabaa Efficiency Index ($\eta^{Nabaa}$)

The Nabaa Efficiency Index ($\eta^{\mathrm{Nabaa}}$) is designed to measure plan quality by combining the (i) geometric conformity of the high‐dose region to the target volume and (ii) the efficiency with which the delivered absorbed dose is confined to that target, rather than spilling into surrounding tissue. The index is dimensionless and independent of prescription dose, enabling comparison across modalities.

- **Mean Dose to Target Volume**

Let D(r) represent the mean dose that was computed directly from the treatment planning system as standard DVH‐derived. The first parameter in the equation is the mean dose that reached the target volume as follows:

(1) $${\bar{D}}_{TV}=\frac{1}{V_{TV}}\;\int_{V_{TV}}D\left(\vec{r}\right)\;dV$$

This formula represents the mean dose received through the whole target volume, where:

- V = Volume
- ${\bar{D}}_{TV}$ Mean dose $\bar{\left(D\right)}$ to the target volume (TV)
- $\left(\vec{r}\right)$ : position vector in 3D space (x, y, z)

The mean dose was computed directly from the treatment planning system using standard DVH‐derived values. It allows one to measure the mean dose in the target correctly, as presented in Figure 1.

**FIGURE 1:**
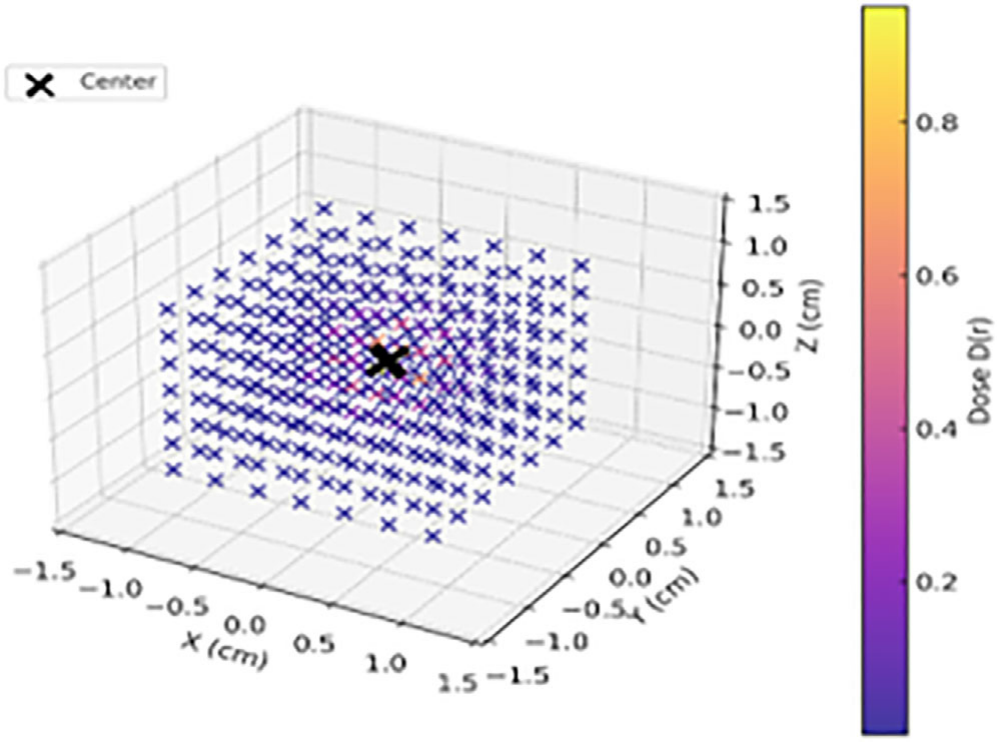
Illustrative graph for mean dose to the target volume.

Then, the equation of the Conformity Index (CI) from the Radiation Therapy Oncology Group (RTOG) ^2^ was reformulated to

(2) $$CI_{x}=\frac{V_{TV}}{V_{PIV_{x}}}$$

- PIV: Prescription Isodose Volume
- $V_{PIV_{x}}$: volume (V) receiving the prescription dose (x). A CI close to 1 indicates high conformity.

Although the conformity index (CI) presents an indication of geometric accuracy, it does not include any data regarding the efficiency of the absorbed dose concentration in the target. To introduce dose efficiency, we define the dose efficiency ratio as follows:

(3) $$DER_{x}=\frac{{\bar{D}}_{TV}}{{\bar{D}}_{PIV_{x}}}$$

Where ${\bar{D}}_{PIV_{x}}$ means the mean dose in the isodose surface under consideration. DER > 1 indicates efficient dose confinement inside the target; DER < 1 indicates dose spill into surrounding tissue.

b. **General Form of the Nabaa Efficiency Index (** $\eta^{\mathrm{Nabaa}}$)

The proposed formulation is conceptually related to previously published efficiency‐based metrics. However, the present work generalizes the efficiency concept by defining a common mathematical framework applicable to different prescription‐isodose levels, particularly 50% and 90%, allowing evaluation of both stereotactic and fractionated radiotherapy plans. Combining the geometric conformity term (Equation 2) and the dose‐efficiency term (Equation 3), the generalized $\eta_{Nabaa}$ expression is defined as:

(4) $$\eta_{Nabaa}=CIXDER=\left(\frac{V_{TV}}{V_{PIV_{x}}}\right)\left(\frac{{\bar{D}}_{TV}}{{\bar{D}}_{PIV_{x}}}\right)$$

This is a dimensionless formulation that combines spatial and dosimetric components of plan quality. A value of $\eta_{Nabaa}$ equal to 1 indicates optimal efficiency, that is, that dose is both geometrically and energetically localized. Values below 1 indicate reduced efficiency due to geometric or dose spill.

c. **Nabaa Efficiency Index** $\eta_{50\%}^{\mathrm{Nabaa}}$

$\eta_{50\%}^{\mathrm{Nabaa}}$ or (NEI_50_) is defined at the 50% prescription isodose. It is intended for situations where assessment of prescription‐dose conformity is the primary objective, irrespective of the treatment delivery technique or fractionation schedule. By combining conformity at the 50% isodose with the ratio of mean target dose to mean dose in the 50% isodose volume, $\eta_{50\%}^{Nabaa}$ reward plans that keep the intermediate dose ‘halo’ tightly confined and penalize spill into surrounding normal tissue. By using equation 4, we proposed the Nabaa Efficiency Index ($\eta_{50\%}^{Nabaa}$), denoted as. Which integrates conformity and dose distribution into the efficiency evaluation:

(5)

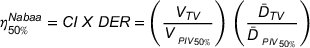

d. **Nabaa Efficiency Index (** $\eta_{90\%}^{\mathrm{Nabaa}}$)

Nabaa Efficiency Index ($\eta_{90\%}^{Nabaa}$) or(NEI_90_) is defined at the 90% prescription isodose. It captures how efficiently the high‐dose region is confined to the target in fractionated and modulated techniques (IMRT/VMAT). A high $\eta_{90\%}^{Nabaa}$ indicates that the prescription dose is both conformal and energetically concentrated in the target, rather than spilling into surrounding tissue. We get another efficacy index at a given prescription isodose volume of 90% as follows:

(6)

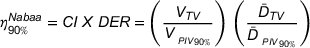




Because both components of Equations 6 and 9 are expressed as ratios of identical physical quantities (volume and dose), the $\eta^{Nabaa}$ is inherently dimensionless:

(7) $$\eta^{Nabaa}=\frac{\left[cm^{3}\right]}{\left[cm^{3}\right]}\;\times \;\frac{\left[Gy\right]}{\left[Gy\right]}=1\mathrm{ss}$$

The dimensionless formulation of $\eta^{Nabaa}$ facilitates comparison across different prescription levels, treatment planning techniques, and planning systems without requiring unit conversion. However, the index does not imply independence from the physical characteristics of the radiation beam. The Nabaa Efficiency Index ($\eta_{90\%}^{Nabaa}$) is inherently dimensionless and modality‐agnostic. The pseudocode to calculate the indices is shown in the box below.

Inputs: Dose grid D(r), target mask TV, isodose masks PIV50, PIV90

Compute volumes: VTV, VPIV50, VPIV90

Compute means: Dbar_TV, Dbar_PIV50, Dbar_PIV90

CI50 = VTV/VPIV50; DER50 = Dbar_TV/Dbar_PIV50; NEI50 = CI50*DER50

CI90 = VTV/VPIV90; DER90 = Dbar_TV/Dbar_PIV90; NEI90 = CI90*DER90

Output: NEI50, NEI90

Table 1 shows the mathematical formulation and variable definitions proposed in this study. All parameters are dimensionless unless otherwise stated.

**TABLE 1:** Notation and symbols used in $\eta^{Nabaa}$ derivation.

Symbol	Definition/Description	Unit	Role in $\eta^{Nabaa}$ Calculation
**D(r)**	Spatial dose distribution as a function of 3D position vector *(x, y, z)*	Gy	A continuous dose field is used for mean dose integration within the target or isodose region.
**V_TV_**	Target volume (tumor or clinical target volume, CTV)	cm^3^	Reference volume for geometric conformity and mean dose to target
**V_PIV_**	Prescription isodose volume (volume enclosed by a defined % of prescription dose)	cm^3^	Used for conformity assessment and dose spill quantification
**PIV_50_ / PIV_90_**	Volumes enclosed by 50% and 90% of the prescription isodose	cm^3^	Define spatial domains for η_50_ and η_90_ computation.
**D_TV_**	Mean dose delivered within the target volume	Gy	Represents dose confinement inside the target
**D_PIV_**	Mean dose within the prescription isodose volume (e.g., 50% or 90%)	Gy	Represents the energy spread in the surrounding dose envelope
**DER**	Dose Efficiency Ratio = $\frac{{\bar{D}}_{\mathrm{TV}}}{{\bar{D}}_{\mathrm{PIV}}}$	(dimensionless)	Quantifies dose localization; > 1 indicates efficient dose confinement.
**CI (RTOG)**	Conformity Index = $\frac{V_{\mathrm{TV}}}{V_{\mathrm{PIV}}}$	—	Measures the geometric coverage of the target by the prescription isodose
$\eta^{Nabaa}$ **(general)**	${\;\eta }^{\mathrm{Nabaa}}$= CI × DER	—	Generalized geometric–dosimetric efficiency measure
$\eta_{50\%}^{Nabaa}$	The Nabaa Efficiency Index evaluated at the 50% isodose level	—	Sensitive to gradient and spill; used in SRS/SBRT applications
$\eta_{90\%\;}^{Nabaa}$	$\eta^{\mathrm{Nabaa}}$ Evaluated at the 90% isodose level	—	Sensitive to high‐dose conformity; used in IMRT/VMAT or hypofractionated plans
**AUC**	Area Under the Receiver Operating Characteristic Curve	—	Quantifies predictive performance of $\eta^{Nabaa}$ vs. reference indices
**TCP / NTCP**	Tumor Control Probability / Normal Tissue Complication Probability	—	Used for future biological validation of $\eta^{Nabaa}$ thresholds

### 2.2 Boundary conditions

Table 2 summarizes limiting behaviors of the index under ideal and non‐ideal conditions, and Table 3 proposes interpretive ranges for clinical use. These interpretive thresholds were empirically derived from the observed distribution of NEI values within the clinical dataset (n = 100) and are intended as preliminary guidance pending external validation. Figure 2 shows the workflow of equations.

**TABLE 2:** Theoretical evaluation of $\eta^{Nabaa}.$

Condition	CI	DER	$\eta^{Nabaa}$	Interpretation
Perfect conformity and ideal dose	1.0	1.0	1.0	Optimal plan efficiency
Perfect conformity with the dose spill	1.0	<1.0	<1.0	Efficient geometry but poor energy use
Imperfect conformity with the ideal dose	<1.0	1.0	<1.0	Accurate energy use but geometric loss
Overconcentration in the target	1.0	>1.0	>1.0	Steep gradient, ideal for SRS
Diffuse low‐dose spread	<1.0	<1.0	≪1.0	Inefficient plan with extensive spill

**TABLE 3:** Theoretical interpretive framework.

$\eta^{Nabaa}$ Range	Efficiency classification	Physical meaning
0.90–1.10	Optimal	Balanced conformity and compactness
0.70–0.89	Acceptable	Minor spill or moderate falloff
0.50–0.69	Suboptimal	Significant dose leakage
< 0.50	Inefficient	Geometrically and dosimetrically poor plan

### 2.3 Analytical behavior

The proposed $\eta^{Nabaa}$ was demonstrated under the simulation of theoretical curves from the behavior of the proposed conformity and dose gradient indices using normalized volume and dose ratios.

Figure 3 shows the comparison of the proposed Nabaa Efficiency Index $\eta^{Nabaa}$ in theory to the quality indices that consider the performance of plans, such as the Radiation Therapy Oncology Group (RTOG) Conformity Index, the Paddick Conformity Index (PCI), and the Gradient Index (GI). Figure 4 is exemplary of a simulated theory behavior and is not from any patient‐specific data. The x‐axis is normalized prescription isodose volume (V_PIV_/V_TV_) in correspondence to the increasing amount of dose spill outside the target, while the y‐axis represents the normalized value of each index. The RTOG and Paddick indices show rapid decrease with isodose expansion, which represents the loss of geometric conformity. The GI, in contrast, increases monotonically through the effect of the growing low dose envelope and stresses the inverse sensitivity to dose compactness. The $\eta_{50\%}^{Nabaa}$ and $\eta_{90\%}^{Nabaa}$ curves are shown to have a smooth and bounded monotonic decrease, which confirmed that the index effectively combines both geometrical and dosimetric efficiency. At unity (V_PIV_/V_TV_ = 1), all indices are at 1.0, representing the ideal plan condition. As the ratio grows to 2.0, $\eta^{Nabaa}$ values fall within a narrower and more physically interpretable range of values than traditional indices, where the rapidly growing saturation in geometrical values can be seen in PCI or in the uncontrolled escalation of GI. The $\eta_{50\%}^{Nabaa}$ exhibit high sensitivity gradients of compute‐dose degradation, which is rather suitable for high‐dose stereotactic treatments, whereas $\eta_{90\%}^{Nabaa}$ exhibits higher smoothness of attenuation gradients closer to the dosage conformity preferences of economy factors of conventional fractionated plans. Overall, the good agreement between the simulated and calculated curves confirms that $\eta^{Nabaa}$ augmenter over a broad range of geometrical conditions retains the dimensional neutrality and produces a balanced reflection of the spatial and dose plan performances.

**FIGURE 2:**
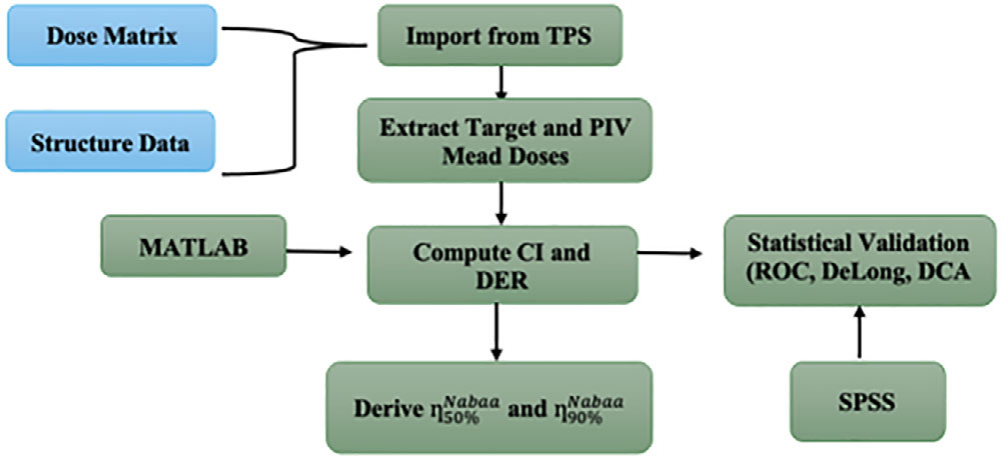
Workflow of index equations.

In a clinical sense, the tendencies in Figure 3 indicate that the Nabaa Efficiency Index ($\eta^{Nabaa}$) is more interpretable and actionable than traditional indices due to its ability to provide a measure of plan quality. High values of $\eta^{Nabaa}$ (> 0.90) are associated with treatment plans that have high conformity and low energy dispersion, which generally implies excellent dose confinement and exposure to low levels of surrounding organs at risk (OARs). Moderate $\eta^{Nabaa}$ values (0.7–0.9) present an acceptable clinical performance but indicate there could be an opportunity to enhance dose compactness or conformity. indicates that the energy is used inefficiently and is likely to cause geometric overexposure, which also requires re‐optimization in the plan. Since $\eta^{Nabaa}$ incorporates space and dosimetric efficiency, it can be used as a single decision‐support measurement in both fractionated and stereotactic planning processes. The difference between $\eta_{50}^{Nabaa}$ and $\eta_{90}^{Nabaa}$ also has clinical implications: $\eta_{50}^{Nabaa}$ useful in ablative SRS/SBRT cases where steep dose falloff is necessary, whereas $\eta_{90}^{Nabaa}$ is more useful in IMRT or VMAT plans where steep falloff is not necessary, like cases in the abdominal area. $\eta^{Nabaa}$ could be added as an optimization criterion or automatic scoring variable into future clinical implementations during treatment planning systems to objectively seek maximum therapeutic efficacy and minimize inter‐planner variance. These features make $\eta^{Nabaa}$ a potential universal scale of contemporary quantitative, cross‐modality plan evaluation in radiotherapy. Table 4 summarizes the advantage of $\eta^{Nabaa}$ compared to the other evaluation indexes.

**TABLE 4:** The important conceptual differences between the $\eta^{Nabaa}$ current plan and quality indices.

Metric	Evaluates	Limitations	$\eta^{Nabaa}$ integration advantage
**RTOG conformity index**	Volume conformity only	Ignores dose magnitude and falloff	$\eta^{Nabaa}$ adds energy weighting through the mean dose ratio
**Paddick conformity index (PCI)**	Geometric overlap	No dose weighting; static geometry	$\eta^{Nabaa}$ generalizes with dynamic dose–volume coupling
**Paddick efficiency index (** $\eta_{50\%}^{Paddick}$)	Geometric compactness	Designed primarily for stereotactic applications	$\eta^{Nabaa}$ generalized to multiple prescription isodose levels
**Gradient index (GI)**	Falloff steepness	No internal dose assessment	$\eta_{50\%}^{Nabaa}$ embeds falloff and mean dose correlation
**Homogeneity index (HI)**	Target dose uniformity	Ignores spatial distribution	$\eta^{Nabaa}$ Couples internal and external dose behavior
$\eta^{Nabaa}$ **(Proposed)**	Combined conformity and efficiency	—	Unified geometric–dosimetric model applicable to all modalities

### 2.4 Applicability across modalities

The dual formulation of $\eta^{Nabaa}$ allows its application across both high‐dose single‐fraction and multi‐fraction conventional therapies.

- In SRS and SBRT, where precision and rapid dose falloff are essential, it $\eta_{90\%}^{Nabaa}$ acts as a sensitive indicator of spatial compactness and gradient sharpness. Its inverse dependence on the 50% isodose volume penalizes dose leakage into normal tissue, making it directly relevant to minimizing radiation necrosis risk in brain or lung lesions.
- In IMRT and VMAT, where dose homogeneity and prescription adherence dominate, $\eta_{90\%}^{Nabaa}$ quantifies how efficiently the prescribed dose is confined to the clinical target volume (CTV) while maintaining overall plan smoothness.

Thus, $\eta_{50\%}^{Nabaa}$ and $\eta_{90\%}^{Nabaa}$ together form a dual‐axis evaluation system, enabling theoretical comparison between radically different treatment philosophies—ablative stereotactic versus fractionated conventional—within a single mathematical language.

### 2.5 Comparison to Paddick efficiency index

The Paddick efficiency index ($\eta_{50\%}^{Paddick}$) introduced an important efficiency‐based extension for stereotactic plan assessment. The present framework builds upon this concept by expressing efficiency within a generalized formulation that can be evaluated at different prescription‐isodose levels, including the clinically relevant 90% prescription isodose.

This limitation is addressed by a Nabaa Efficiency Index ($\eta^{Nabaa}$) which incorporates a dosimetric term—the Dose Efficiency Ratio (DER = mean target dose/mean isodose envelope dose). Consequently, $\eta^{Nabaa}$ quantifies both geometric conformity and dosimetric localization, with $\eta_{50\%}^{Nabaa}$ reflecting intermediate‐dose gradient efficiency and $\eta_{90\%\;}^{Nabaa}$ representing high‐dose conformity efficiency across modalities.

### 2.6 Workflow

The analytical workflow expressions of $\eta_{50\%\;}^{Nabaa}$ and $\eta_{90\%}^{Nabaa}$ were evaluated both theoretically and clinically, as summarized in the following results.

### 2.7 Clinical dataset and validation

#### 2.7.1 Study design

A retrospective dosimetric validation was performed on 100 stereotactic plans generated between June and August 2025 at two centers: Warith International Cancer Institute (WICI), Karbala, and the Gamma Knife Center, Ghazi Alharriri Hospital, Iraq. The ethical approval consent for the patient was signed at the Warith International Cancer Institute (WICI) in Karbala, Iraq, and all participants provided informed consent prior to their inclusion in the study. The Human Research Ethics Committee—Jabir Ibn Hayyan University—Faculty of Medical Sciences (HREC‐JMU‐FMS) approved the study with code: (NO:00006‐DATE:2/11/2025).

#### 2.7.2 Cohort description

Eligible patients were adults (≥18 years) diagnosed with multiple brain metastases and an ECOG performance status ≤2. Exclusion criteria included incomplete imaging, prior cranial irradiation, or missing dose–volume data. The final dataset included 50 Gamma Knife Icon (GK‐SRS) and 50 linac‐based SRS‐VMAT treatment plans.

#### 2.7.3 Imaging and simulation

CT simulations were performed using SOMATOM Confidence® (Siemens, Syngo CT VB10A) with 1.25 mm slice thickness, a 512 × 512 matrix, and a 35 cm field of view. MRI datasets were co‐registered for contouring the target and organs‐at‐risk (OARs).

#### 2.7.4 Treatment planning

It was performed using two modalities: The first modality consisted of Gamma Knife plans, created using Leksell Gamma Knife Icon (Elekta, Sweden), while the second consisted of SRS‐VMAT plans generated in Eclipse™ v16 (Varian Medical Systems, Nederland B.V., Inc.). All patients received a prescription of 30 Gy in three fractions, normalized according to institutional protocols. Indices were calculated for each plan.

Endpoints included comparison of $\eta_{50\%\;}^{Nabaa}$, $\eta_{90\%\;}^{Nabaa}$, and η_p_ values between modalities (GK‐SRS vs. SRS‐VMAT), and assessment of discrimination of “optimal” plans (CI_90_ ≥ 0.70) using ROC/AUC analysis. And to evaluate net clinical benefit via Decision Curve Analysis (DCA).

Using the dataset and workflow, we evaluated the performance of $\eta_{50\%\;}^{Nabaa}$, $\eta_{90\%\;}^{Nabaa}$, and the Paddick Efficiency Index ($\eta_{50\%}^{Paddick}$) across 100 stereotactic treatment plans—50 Gamma Knife (GK‐SRS) and 50 linac‐based SRS‐VMAT. Each plan was verified for contour and dose‐volume integrity before index computation.

#### 2.7.5 Analysis workflow

**Workflow**: For each plan, volumes (TV and PIV at selected isodose levels) and mean doses (DTV and DPIV) were extracted from the treatment planning system. NEI50 and NEI90 were computed and compared with established indices.

**Statistical analysis**. Analyses were performed using SPSS v29 (IBM). Continuous variables were expressed as mean ± SD after Shapiro–Wilk normality testing, and categorical variables as frequencies and percentages. Between‐group comparisons used one‐way ANOVA. ROC/AUC (95% CI) quantified discriminative ability, with Youden's J identifying optimal cut‐offs.

AUCs were compared with CI estimation using bootstrap resampling (*n* = 1,000) by applying DeLong tests. Multivariable logistic regression was used with clinical optimality as the dependent variable and controlled for modality. The calibration was checked using the Hosmer‐Lemeshow test and calibration plots. DCA examined the clinical net benefit for threshold probabilities (0.30‐0.70). Significance was set at p ≤ 0.05.

$\eta^{Nabaa}$ computed based on above Equations using Python version 3.12 and for statistics using SPSS version 29, the version‐controlled scripts and fixed random seeds for reproducibility. An overview of a schematic for the entire validation process is shown in Figure 2. The validated dataset was thus able to enable quantitative assessment of $\eta_{50\%\;}^{Nabaa}$ and $\eta_{90\%\;}^{Nabaa}$ across modalities under real‐world stereotactic planning conditions. The subsequent section presents the clinical validation results.

**FIGURE 3:**
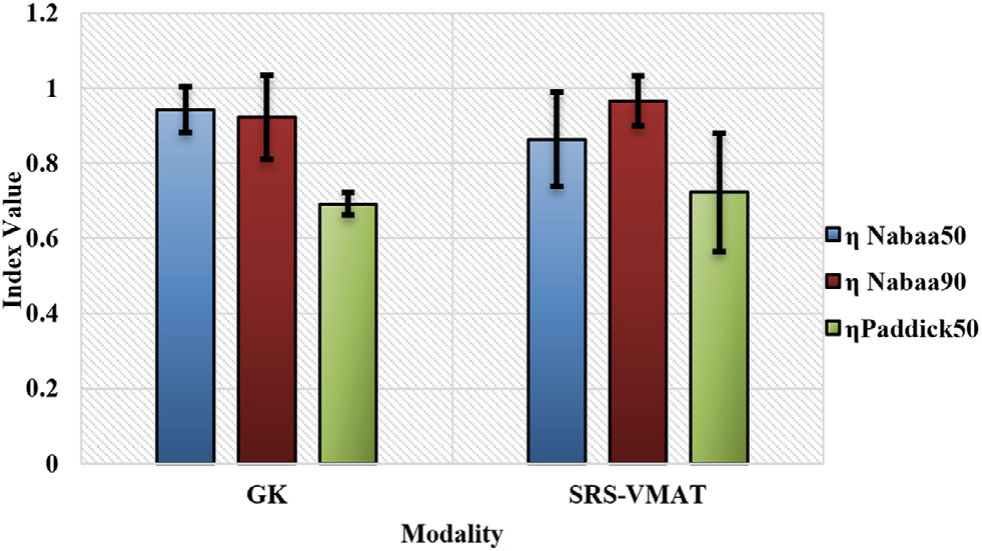
Comparison between the radiotherapy modalities concerning the efficiency indexes.

## 3 RESULTS

### 3.1 Clinical validation

Group comparisons, correlation analyses, and ROC‐based discrimination tests were then conducted to determine how effectively $\eta^{Nabaa}$ reflected plan quality relative to established indices.

### 3.2 Efficiency indices

As shown in Table 5 and Figure 3, Gamma Knife plans exhibited significantly higher $\eta_{50\%}^{Nabaa}$ (0.94 ± 0.06) than SRS‐VMAT (0.86 ± 0.12), demonstrating superior intermediate‐dose control and spatial energy economy. No significant differences were observed for $\eta_{90\%}^{Nabaa}$ (*p* = 0.65) or $\eta_{50\%}^{\mathrm{Paddick}}$ (*p* = 0.37), indicating similar high‐dose conformity between modalities despite distinct delivery mechanics

**TABLE 5:** Efficiency index comparison between multiple metastatic patients treated with GK versus SRS‐VMAT.

Index	GK	SRS‐VMAT	*p‐value*
$\eta_{50\%}^{\mathrm{Nabaa}}$	0.94 ± 0.06	0.86 ± 0.12	≤0.0001
$\eta_{90\%}^{\mathrm{Nabaa}}$	0.92 ± 0.11	0.97 ± 0.06	0.6517
$\eta_{50\%}^{\mathrm{Paddick}}$	0.70 ± 0.02	0.72 ± 0.15	0.3706
***p‐value***	≤0.0001	≤0.0001	

**FIGURE 4:**
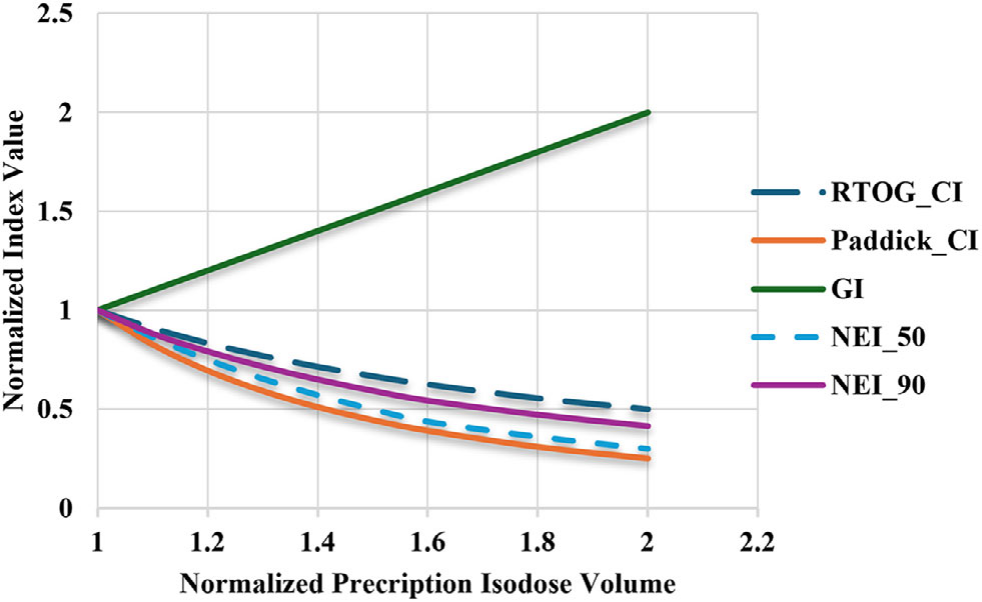
The theoretical comparison between $\eta^{Nabaa}$ and other indices.

### 3.3 Predictive performance and clinical utility

The discrimination analysis in Table 6 and Figure 5 demonstrated clear differences in predictive performance between the $\eta^{Nabaa}$ and the $\eta_{50\%}^{Paddick}$ across treatment modalities. $\eta_{90\%}^{Nabaa}$ achieved AUC 0.902 (GK‐SRS) and 0.977 (VMAT‐SRS) for discriminating clinically “optimal” plans (PCI_90_ ≥ 0.70), clearly outperforming $\eta_{50\%}^{Nabaa}$ and η_p_ in both modalities.  In pooled analysis (*n* = 100), $\eta_{90\%}^{\mathrm{Nabaa}}$ kept very high discrimination (AUC 0.941) with sensitivity 0.863 and specificity 0.931 at a cut‐off of 0.866.

**TABLE 6:** The discrimination analysis as a predictive performance between the Nabaa Efficiency Index ($\eta^{\mathrm{Nabaa}}$) and the Paddick Efficiency Index ($\eta_{50\%}^{Paddick}$) across treatment modalities.

Modality	Metric	AUC	Sensitivity	Specificity	Cut‐off
GK‐SRS	$\eta_{50\%}^{\mathrm{Nabaa}}$	0.332	0.025	1.000	1.002
GK‐SRS	$\eta_{90\%}^{\mathrm{Nabaa}}$	0.902	0.900	0.800	0.871
GK‐SRS	$\eta_{50\%}^{\mathrm{Paddick}}$	0.475	1.000	0.200	0.475
SRS‐VMAT	$\eta_{50\%}^{\mathrm{Nabaa}}$	0.604	0.719	0.556	0.830
SRS‐VMAT	$\eta_{90\%}^{\mathrm{Nabaa}}$	0.977	0.938	0.944	0.823
SRS‐VMAT	$\eta_{50\%}^{\mathrm{Paddick}}$	0.58	0.469	0.778	0.747
All	$\eta_{50\%}^{\mathrm{Nabaa}}$	0.539	0.808	0.379	0.830
All	$\eta_{90\%}^{\mathrm{Nabaa}}$	0.941	0.863	0.931	0.866
All	$\eta_{50\%}^{\mathrm{Nabaa}}$	0.537	0.671	0.5	0.646

**FIGURE 5:**
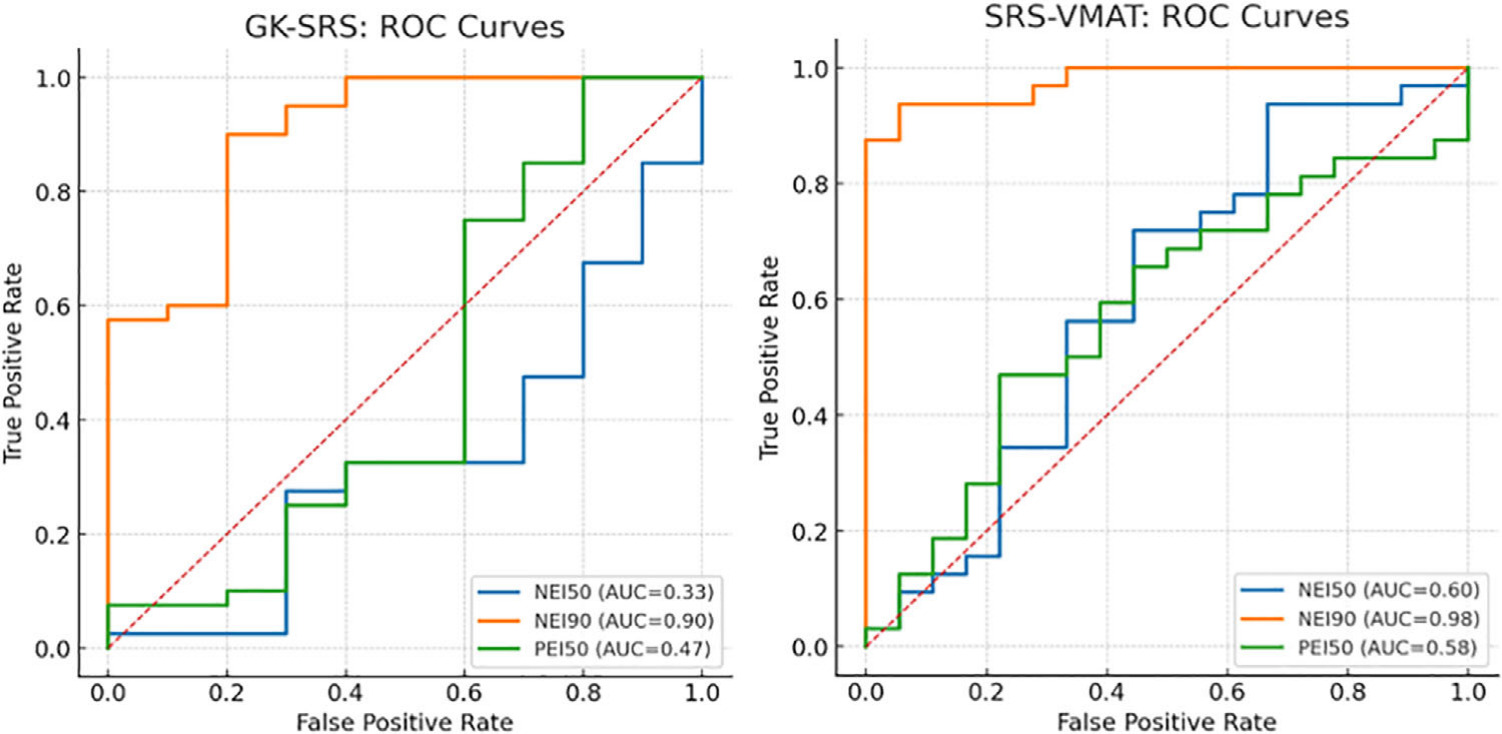
The ROC analysis between the indices across treatment modalities.

The comparison between Gamma Knife and linac‐based stereotactic radiotherapy in the present study was intended to demonstrate the applicability of the proposed $\eta^{Nabaa}$ framework across different treatment delivery platforms rather than to establish the clinical superiority of one technique over another. Since each modality is selected according to specific clinical indications, target characteristics, and institutional resources, direct comparison between techniques should not be interpreted as a plan‐selection exercise. Instead, the present findings demonstrate that the proposed framework can be applied consistently across different stereotactic planning approaches. We agree that the most clinically relevant validation of $\eta^{Nabaa}$ will involve comparison of multiple competing plans generated for the same patient using the same treatment technique. Such within‐technique plan‐selection studies constitute the next stage of this work and will evaluate the ability of $\eta^{Nabaa}$ to assist treatment planners in selecting the optimal plan among clinically acceptable alternatives.

In Figure 6, the decision curve analysis (DCA), the $\eta_{90\%}^{Nabaa}$ consistently demonstrated superior clinical utility compared to the $\eta_{50\%}^{Paddick}$, particularly in the SRS‐VMAT cohort. Decision Curve Analysis showed higher net clinical benefit when selecting plans using $\eta_{90\%}^{Nabaa}$ rather than $\eta_{50\%}^{Paddick}$, especially in VMAT‐SRS between threshold probabilities 0.40–0.60. Comparative Strengths, Limitations, and Optimal Clinical Applications shown in Table 7.

**FIGURE 6:**
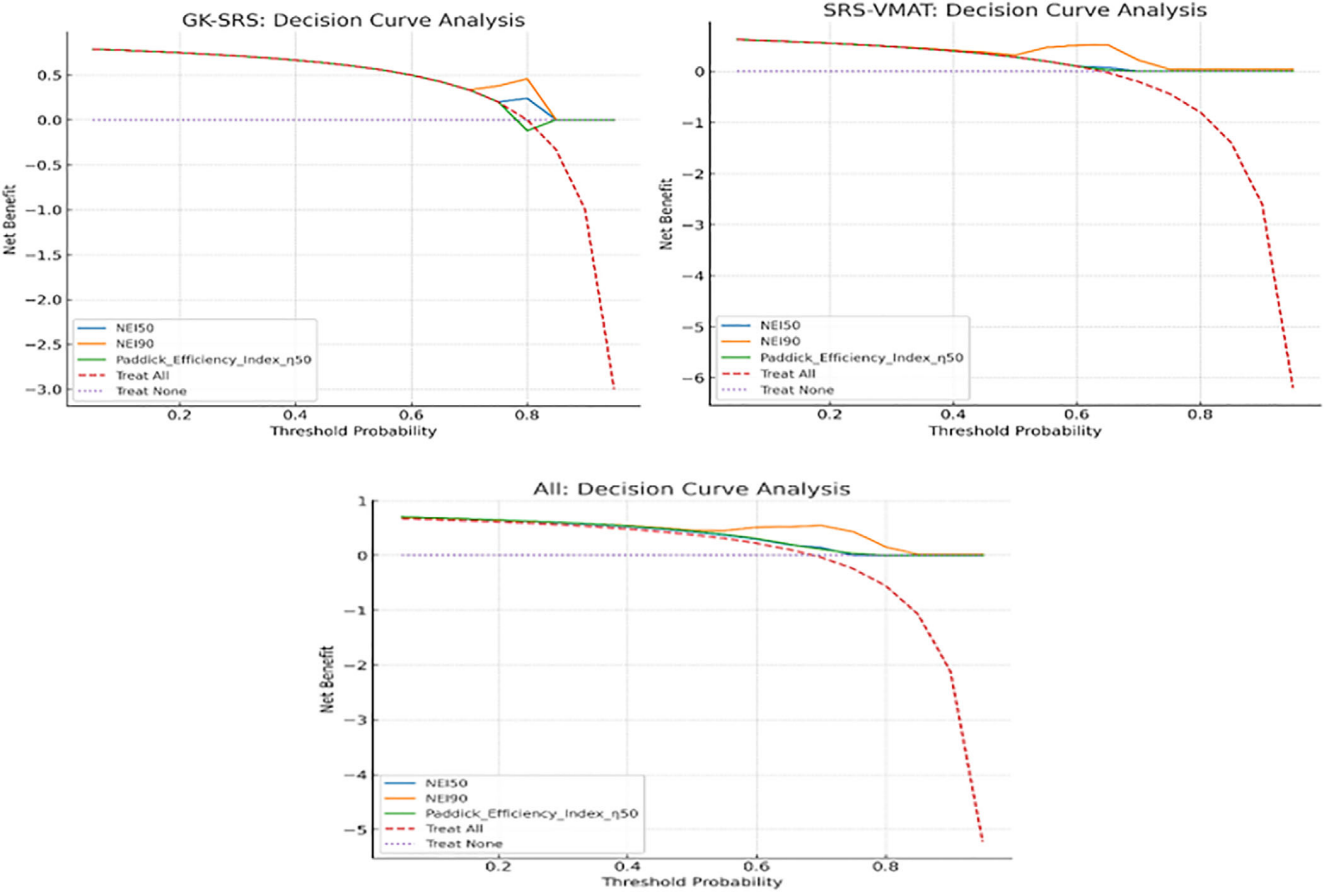
The decision curve analysis (DCA) between the indices across treatment modalities.

**TABLE 7:** Comparative strengths, limitations, and optimal clinical applications of $\eta_{50\%}^{Nabaa}$, $\eta_{90\%}^{Nabaa}$, and Paddick Efficiency Index ($\eta_{50\%}^{Paddick}$).

Index	Primary strength	Limitations	Ideal clinical use case
$\eta_{50\%}^{\mathrm{Nabaa}}$	Balanced assessment between target coverage and dose spill; interpretable threshold (≈1.0 = optimal balance)	Smaller AUC gains vs. $\eta_{90\%}^{Nabaa}$; may be less sensitive for detecting high‐concentration plans.	Cases where both central dose and peripheral sparing are equally important; toxicity‐sensitive planning
$\eta_{90\%}^{\mathrm{Nabaa}}$	Strongest predictor of optimal plan quality in SRS‐VMAT (ΔAUC = 0.40 vs $\eta_{50\%}^{Paddick}$, p < 0.001); high sensitivity/specificity; robust calibration and DCA	Slightly less intuitive interpretation; minimal AUC gain in GK‐SRS	Linac‐based SRS, high‐precision stereotactic boosts, automated plan ranking
$\eta_{50\%}^{\mathrm{Paddick}}$	Established benchmark; familiar with the literature	Less discriminative than $\eta_{90\%}^{\mathrm{Nabaa}}$ (especially in SRS‐VMAT); weaker calibration; lower net benefit in DCA	Historical comparisons are a baseline for evaluating new indices.

### 3.4 Correlation of Nabaa efficiency index

The correlation analysis shown in Table 8 demonstrated that the Nabaa Efficiency Index ($\eta^{\mathrm{Nabaa}}$) exhibited strong and consistent associations with established plan quality indices. $\eta_{50\%}^{Nabaa}$ showed a strong positive correlation with PCI_50_ (*r* = 0.723, *p* < 0.001), indicating that both metrics primarily quantify geometric conformity at the intermediate‐dose level. Its weak relationship with PCI_90_ (*r* = 0.108, *p* = 0.28) confirmed that $\eta_{50\%}^{Nabaa}$ behaves independently from high‐dose conformity, reflecting its distinct sensitivity to dose gradient and spill control. A moderate correlation with the Homogeneity Index (HI) (*r* = 0.358, *p* < 0.001) suggests a limited overlap in describing dose uniformity, while its negligible correlation with the $\eta_{50\%}^{Paddick}$ (*r* = –0.006, *p* = 0.95) reinforces that $\eta_{50\%}^{Nabaa}$ captures a unique efficiency characteristic not represented by conventional geometric metrics.

**TABLE 8:** Correlation of Nabaa Efficiency Index ($\eta^{\mathrm{Nabaa}}$) with other indices.

Comparison	(*r)*	*p*‐value
$\eta_{50\%}^{\mathrm{Nabaa}}$ **vs. PCI_50_**	**0.723**	< 0.0001
$\eta_{50\%}^{\mathrm{Nabaa}}$ **vs. PCI_90_**	0.108	0.28
$\eta_{50\%}^{\mathrm{Nabaa}}$ **vs. HI**	0.358	< 0.001
$\eta_{50\%}^{\mathrm{Nabaa}}$ **vs**. $\eta_{50\%}^{Paddick}$	−0.006	0.95
$\eta_{90\%}^{Nabaa}$ **vs. PCI_50_**	0.000	0.99
$\eta_{90\%}^{Nabaa}$ **vs. PCI_90_**	**0.984**	< 0.0001
$\eta_{90\%}^{Nabaa}$ **vs. HI**	0.112	0.27
$\eta_{90\%}^{Nabaa}$ **vs**. $\eta_{50\%}^{Paddick}$	0.289	0.003

Similarly, $\eta_{90\%}^{Nabaa}$ demonstrated an extremely strong linear correlation with PCI_90_ (*r* = 0.984, *p* < 0.001), confirming that both parameters reflect high‐dose prescription conformity. In contrast, $\eta_{90\%}^{Nabaa}$ was uncorrelated with PCI_50_ (*r* = 0.000, *p* = 0.99) and weakly associated with HI (*r* = 0.112, *p* = 0.27), indicating that it remains largely independent of dose homogeneity. A mild positive relationship with the $\eta_{50\%}^{Paddick}$ (*r* = 0.289, *p* = 0.003) suggests that $\eta_{90\%\;}^{Nabaa}$ shares some geometric dependency with existing indices but incorporates a more explicit dosimetric weighting. Collectively, these findings highlight that $\eta_{50\%}^{Nabaa}$ serves as a gradient‐efficiency indicator, whereas $\eta_{90\%}^{Nabaa}$ represents prescription‐dose conformity. Together, they form a dual and complementary framework for evaluating plan quality across modalities.

The observed behavior of $\eta^{Nabaa}$ across modalities forms the basis for the following discussion on its novelty and clinical relevance

## 4 DISCUSSION

This study introduces the Nabaa Efficiency Index ($\eta^{Nabaa}$), a unified plan‐quality metric. Conventional indices such as CI, HI, and DVH‐based measures describe individual aspects of plan quality (conformity, uniformity, or dose distribution), but none integrate geometric conformity and dose localization in a single, dimensionless index. $\eta^{Nabaa}$ addresses this by combining both components into one interpretable measure that can be applied across modalities

### 4.1 Nabaa efficiency index at 50% isodose ($\eta_{50\%}^{Nabaa}$)

This $\eta_{50\%}^{Nabaa}$ is particularly relevant in the context of stereotactic radiosurgery (SRS) where large dose gradients and low dose spillage are paramount because of the close contact of organs at risk (OARs). It defined as the product of 50% Conformity Index and Dose Efficiency Ratio within 50% isodose envelope $\eta_{50\%}^{Nabaa}$ is the measure of efficiency in the concentration of energy on the target without punishing medium dose spillage. Plans with broad intermediate‐dose spread show lower $\eta_{50\%}^{Nabaa}$, reflecting geometric and dosimetric inefficiency.

This behavior is supported by clinical evidence. These studies by Minniti et al.^4^ and Vogelbaum et al.^5^ show that the risk of radionecrosis in brain metastasis SRS is correlated with the excessive spread of intermediate dose (e.g., large V12 volumes). Similarly, Lippitz et al.^6^ demonstrated that the steep dose falloff plans enhanced neurological sparing in functional radiosurgery.

### 4.2 Nabaa efficiency index at 90% isodose ($\eta_{90\%}^{Nabaa}$)

The $\eta_{90\%}^{Nabaa}$ is optimized for VMAT/IMRT‐style delivery, where the clinical goal is high‐dose conformity to the target while maintaining acceptable uniformity. It captures how efficiently prescription‐absorbed dose is confined to the target volume rather than deposited in surrounding tissue.

### 4.3 Integration and comparative

The $\eta^{Nabaa}$ indices, including $\eta_{50\%}^{Nabaa}$ and $\eta_{90\%}^{Nabaa}$, bridges stereotactic and conventional paradigms in a single, normalized measure. Prior work shows that normalized plan‐quality metrics improve reproducibility and inter‐planner consistency; $\eta^{Nabaa}$ is well‐suited for automated plan ranking, knowledge‐based planning, and a QA dashboard. Unlike previously published efficiency metrics that were developed for stereotactic applications, the proposed framework extends the efficiency formulation to additional prescription‐isodose levels while preserving a simple dimensionless mathematical structure.

Studies by Jones et al.^7^ and Momin et al.^8^ demonstrate that the addition of normalized plan‐quality metrics increases reproducibility and consistency. In this regard, $\eta^{Nabaa}$ may be utilized as a universal efficiency‐based plan optimization and standardization of QA across institutions

### 4.4 Clinical relevance

High $\eta^{Nabaa}$ values (≥ 0.9) represent high conformity and energy confinement, which is associated with lower exposure of OAR and better therapeutic ratios. Intermediate values (0.7 to 0.9) indicate acceptable efficiencies, whereas values of less than 0.7 are indicative of possible geometrical or dosimetric inefficiencies and that need plan refinement. These interpretive ranges are in line with the literature in SRS and SBRT regarding plan‐quality classifications.^9^, ^10^

Subsequent research ought to benchmark $\eta^{Nabaa}$ to biological surrogates including Tumor Control Probability (TCP) and Normal Tissue Complication Probability (NTCP) using approaches suggested by van Herk et al.^11^ and Alexandrian et al.^12^ After its validation, $\eta^{Nabaa}$ might become a cross‐platform reference metric that would look to align plan evaluation across the technologies and institutions.

### 4.5 Efficiency indices

GK demonstrated a significantly higher $\eta_{50\%}^{\mathrm{Nabaa}}$ than SRS‐VMAT (0.86 ± 0.12; p ≤ 0.0001), indicating superior control of the intermediate‐dose region. This reflects GK's multiple collimated Co‐60 beams producing steep dose gradients, keeping the 50% isodose tightly confined to the target. SRS‐VMAT, although conformal, generates a broader intermediate‐dose spread due to cumulative entrance/exit dose and MLC modulation. $\eta_{50\%}^{Nabaa}$, which penalizes expansion of PIV50, effectively captures this distinction.

$\eta_{90\%}^{\mathrm{Nabaa}}$ showed no significant difference (0.92 ± 0.11 vs. 0.97 ± 0.06; *p* = 0.6517), confirming that both modalities achieve similar high‐dose conformity. $\eta_{50\%}^{Paddick}$ also revealed no significant variation (0.70 ± 0.02 vs. 0.72 ± 0.15; *p* = 0.3706), reflecting its lower sensitivity to intermediate dose spread. Clinically, $\eta_{50\%}^{Nabaa}$ is more informative for detecting gradient control and limiting dose spill, particularly important for lesions near critical structures, while $\eta_{90\%}^{Nabaa}$ affirms equivalent high‐dose coverage.

These findings align with previous dosimetric studies. Potrebko et al.^13^ reported that GK provides similar conformity to single‐isocenter VMAT but with reduced low/intermediate‐dose spread, consistent with the $\eta_{50\%}^{Nabaa}$ advantage. Thomas et al.^14^ observed comparable V12 and fall‐off between multi‐arc VMAT and GK, supporting the non‐significant $\eta_{90\%}^{Nabaa}$ difference.

### 4.6 Predictive performance and clinical utility

This study provides direct evidence that the Nabaa Efficiency Index at the 90% isodose level ($\eta_{90\%\;}^{Nabaa}$, or NEI_90_) is a more powerful and clinically relevant discriminator of stereotactic plan quality than both the 50% formulation ($\eta_{50\%\;}^{Nabaa}$, NEI_50_) and the widely used Paddick Efficiency Index ($\eta_{50\%}^{Paddick}$). Across Gamma Knife (GK‐SRS) and linac‐based SRS‐VMAT, $\eta_{90\%}^{Nabaa}$ achieved the highest discriminative performance, with ROC AUC values of 0.902 for GK‐SRS and 0.977 for SRS‐VMAT, representing absolute gains of 0.427 and 0.397, respectively, over $\eta_{50\%}^{Paddick}$. In pooled analysis (*n* = 100), $\eta_{90\%}^{Nabaa}$ maintained an AUC of 0.941 with high sensitivity and specificity at the optimal cut‐off, demonstrating robustness and reproducibility across delivery platforms.

Although its AUC was lower than $\eta_{90\%}^{Nabaa}$, $\eta_{50\%}^{Nabaa}$ remains clinically important. $\eta_{50\%}^{Nabaa}$ balances target coverage with control of the intermediate‐dose “halo,” making it valuable in toxicity‐sensitive scenarios where both ablative coverage and peripheral sparing are critical. By penalizing expansion of the 50% isodose volume (PIV_50_), $\eta_{50\%}^{Nabaa}$ is sensitive to dose spill into surrounding normal tissue and can highlight plans at higher risk of unwanted normal‐tissue exposure near organs‐at‐risk. This behavior is not fully captured by purely geometric indices such as $\eta_{50\%}^{Paddick}$.

Decision Curve Analysis (DCA) confirmed that $\eta_{90\%}^{Nabaa}$’s statistical advantage also translates into practical clinical benefit. For SRS‐VMAT, $\eta_{90\%}^{Nabaa}$ provided the greatest net clinical benefit across threshold probabilities of 0.30–0.70, with a clear advantage in the 0.40–0.60 decision range, which is typically where planners choose between candidate plans. In GK‐SRS, $\eta_{90\%}^{Nabaa}$ also maintained an advantage in the 0.45–0.60 range, and pooled results reflected the same pattern, supporting its generalizability. In summary, $\eta_{90\%}^{Nabaa}$ excels at identifying high‐quality plans based on prescription‐dose conformity and dose efficiency, while $\eta_{50\%}^{Nabaa}$ provides a complementary sensitivity of intermediate‐dose spillage and gradient against, enriching the analytical tool kit that is available for evaluating the stereotactic planning. When used in conjunction, the two formulations combine into a whole and outcome‐ and decision‐based framework which goes beyond standard indices in terms of predictive accuracy, and ultimately by stimulating safer, more uniform decision‐making in radiosurgery.

Unlike simple geometric combinations, the proposed index introduces a dose‐weighted efficiency term (DER), which transforms the conformity assessment into a physically meaningful energy‐density metric. This extends beyond existing indices by incorporating spatial‐dose coupling rather than purely volumetric overlap, allowing for a more accurate representation of how dose is distributed in three‐dimensional space.

Although the present study demonstrates the mathematical formulation and cross‐platform applicability of $\eta^{Nabaa}$, future investigations should focus on same‐patient, same‐technique comparisons involving multiple competing treatment plans and independent expert evaluation to further establish its clinical decision‐support value.

## 5 CONCLUSION

The Nabaa Efficiency Index $\left(\eta^{Nabaa}\right)$ provides a unified, dimensionless framework integrating geometric conformity and dosimetric efficiency for radiotherapy plan evaluation. In a cohort of 100 plans, $\eta_{90\%}^{Nabaa}$ demonstrated superior discrimination—especially for SRS‐VMAT. While $\eta_{50\%}^{Nabaa}$ provided complementary sensitivity to gradient control. Given its simplicity, physical interpretability, and modality‐agnostic design, $\eta^{Nabaa}$ is ready for integration into automated planning and QA workflows as a standardized benchmark alongside existing indices. The aim of the new index is to reduce the time and efforts for physicists to perform clinical decision‐making by providing a quick, quantitative QA surrogate. Through its combination of geometric conformity and dose‐efficiency, NEI gives an early assessment of plan quality instead of the dosimetry and QA processes. Rather than replacing existing conformity metrics, the proposed framework complements them by integrating geometric conformity and dose‐efficiency within a single mathematical formulation.

## AUTHOR CONTRIBUTIONS

**Nabaa Mohammed Alsiraj**: Conceptualization of the NEI framework; theoretical development; manuscript drafting; figure preparation and overall coordination of the work. **Haydar Hamza Alabedi**: Critical review of scientific input on radiotherapy plan‐evaluation metrics and refinement of the theoretical sections. **Mustafa Salih Almusawi**: Independent assessment of the index structure; feedback on methodological clarity; and technical review of dosimetric components. **Moneer K. Faraj**: Clinical physics perspective; evaluation of the potential applicability of the NEI within current planning practices; and editorial suggestions. **Rozilawati Ahmad (Main Supervisor)**: Supervision; guidance on manuscript structure; oversight of the theoretical framing; and final approval of the submitted version. **Sura Abdulkareem Madlool Alzuabidi**: Editing; reviewing.

## CONFLICT OF INTEREST STATEMENT

The authors declare no conflicts of interest.

## ETHICS STATEMENT

The Research Ethics Committee of The National University of Malaysia (RECUKM) approved the study under Ethics Reference No. JEP‐2026‐154 on April 9, 2026.

## GENERATIVE AI DISCLOSURE

Generative artificial intelligence tools were used solely for language editing and improvement of clarity. All scientific content, mathematical formulations, statistical analyses, interpretation of results, and final manuscript content were reviewed and verified by the authors, who take full responsibility for the work.

## Data Availability

The data that support the findings of this study are available from the corresponding author upon reasonable request, subject to institutional and ethical restrictions.
